# Prof. Friedrich Loeffler

**Published:** 1915-05

**Authors:** 


**Prof. Friedrich Loeffler,** who with Klebs, discovered the diphtheria bacillus, died in Berlin, April 9, aged 63.

